# Prognostic and predicted significance of Ubqln2 in patients with hepatocellular carcinoma

**DOI:** 10.1002/cam4.3040

**Published:** 2020-04-15

**Authors:** Yuan‐Deng Luo, Hong‐Qiang Yu, Xiao‐Yu Liu, Deng Huang, Hai‐Su Dai, Lei Fang, Yu‐Jun Zhang, Jie‐Juan Lai, Yan Jiang, Ling Shuai, Lei‐Da Zhang, Geng Chen, Ping Bie, Chuan‐Ming Xie

**Affiliations:** ^1^ Key Laboratory of Hepatobiliary and Pancreatic Surgery Institute of Hepatobiliary Surgery Southwest Hospital Third Military Medical University (Army Medical University) Chongqing China; ^2^ School of Medicine the Southern University of Science and Technology Shenzhen Guangdong China; ^3^ Institute of Hepatobiliary Surgery The Third Affiliated Hospital of Chongqing Medical University Chongqing China

**Keywords:** CTNNB1, HCC, nomogram, overall survival, Ubqln2

## Abstract

**Purpose:**

Hepatocellular carcinoma (HCC) is a common malignant cancer and the third leading cause of death worldwide. The molecular mechanism of HCC remains unclear. Recent studies have demonstrated that the ubiquitin‐proteasome system (UPS) is associated with HCC. Ubqln2, a member of the UPS, is abnormally expressed in HCC. However, whether Ubqln2 is associated with HCC prognosis remains unknown.

**Patients and Methods:**

We analyzed the associations between overall survival and various risk factors in 355 HCC tissue samples obtained from the Cancer Genomic Atlas (TCGA) database at the mRNA level and in 166 HCC tissue samples from Southwest Hospital at the protein level. qRCR was used to determinate Ubqln2 expression in cancer and noncancerous tissues. The association between Ubqln2 and Ki‐67 was analyzed by immunohistochemistry. The association between Ubqln2 expression and survival was analyzed using Kaplan‐Meier curve and Cox proportional hazards models. A nomogram was used to predict the impact of Ubqln2 on prognosis. Mutated genes were analyzed to determine the potential mechanism.

**Results:**

Ubqln2 highly expressed in HCC tissues. The Ubqln2 mRNA level had significant relations with UICC tumor stage (*P* = .022), UICC stage (*P* = .034) and resection potential (*P* = .017). Concordantly, the Ubqln2 protein was closely associated with tumor size (*P* = .005), UICC stage (*P* = .012), and recurrence (*P* = .009). Ubqln2 was highly expressed in HCC and positively associated with poor survival. The nomogram precisely predicted the prognosis of HCC patients with high or low Ubqln2 expression. A genomic waterfall plot suggested that Ubqln2 expression was closely associated with mutated CTNNB1.

**Conclusion:**

Our findings reveal that Ubqln2, an independent risk factor for HCC, is a potential prognostic marker in HCC patients. Ubqln2 expression is positively associated with mutated CTNNB1.

## Graphical abstract

Ubqln2 is abnormally expressed in HCC but its role remains unknown. Here, we reported that Ubqln2, an independent risk factor for HCC, is a potential prognostic marker in HCC patients. Ubqln2 expression is positively associated with mutated CTNNB1.

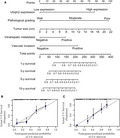

## 1 INTRODUCTION

Among new cancer cases worldwide, liver cancer accounts for more than 840 000 cases every year, and approximately 80%‐90% of these cases are hepatocellular carcinoma (HCC).^1^, ^2^ HCC has been ranked as the sixth most common malignancy and third leading cause of cancer‐related death.^3^ HCC is usually caused by chronic infection with hepatitis B virus (HBV) or hepatitis C virus (HCV), metabolic syndrome related to diabetes and obesity, or alcohol abuse^2^, ^4^ and is regarded as a major cause of death among patients with cirrhosis, and its incidence is speculated to increase in the future.^3^ Although treatment with advanced surgical resection, ablation, new adjuvant therapy or liver transplantation is possible, the 5‐year survival rate of patients with HCC still remains low due to the high frequencies of recurrence and metastasis.^5^, ^6^ Despise the breakthrough invention of sorafenib, an oral multitarget tyrosine kinase inhibitor that has dual antiangiogenic and antiproliferative effects, this chemotherapeutic extends the median overall survival (OS) of patients with advanced‐stage HCC from 7.9 months to only 10.7 months. Moreover, due to sorafenib resistance and adverse events, sorafenib benefits are mostly constrained.^7^, ^8^ Thus, developing novel therapies for HCC remains urgently required.

The modification of proteins via ubiquitylation can regulate most cellular pathways, including those pathways involved in cancer.^9^ The three key enzymes are a ubiquitin‐activating enzyme (E1), ubiquitin‐conjugating enzyme (E2), and ubiquitin ligase (E3), with the addition of the proteasome and a deubiquitinating (DUB) enzyme constituting the ubiquitin‐proteasome system (UPS).^9^, ^10^ The specificity of the UPS is largely dependent on the ~600 E3s that recognize and link their cognate substrates and the level of delivery to the 26S proteasome, as ubiquitylated proteins are directly taken in by the proteasome through its stoichiometric subunits (RPN10 and RPN13) or through loosely associated shuttle factors, which link polyubiquitylated proteins and the proteasome to facilitate degradation.^11^ Ubiqulin (Ubqln) is a family of shuttle factors whose members play roles as adaptors in proteasomal degradation. Humans have four known paralogs of Ubqln, named Ubqln1‐4, as well as Ubqln‐like (UbqlnL).^12^ Ubqln1, Ubqln2, Ubqln4, and UbqlnL are expressed widely, while Ubqln3 is restricted to the testis.^13^

To date, targeting the UPS has been applied to treat HCC with the proteasome inhibitor bortezomib.^14^, ^15^ However, it is still unclear whether the Ubqln shuttle factors can be therapeutic targets for the treatment of HCC. Here, we take advantage of two clinical trial datasets of patients with HCC who underwent surgical resection from the TCGA database and our hospital and report that Ubqln2 is highly correlated with the prognosis of HCC and a potential therapeutic target for the treatment of HCC.

## 2 MATERIALS AND METHODS

### 2.1 Study design and patients

In this retrospective cohort study, we used 166 cases of formalin‐fixed, paraffin‐embedded tumor and adjacent normal tissue samples collected at Southwest Hospital and clinical outcome data from the Clinical Research Center of the Institution of Hepatobiliary Surgery at Southwest Hospital. Data for another 355 cases of HCC were obtained from the Cancer Genomic Atlas (TCGA) database.

All the specimens we obtained from Southwest Hospital met the following criteria: (a) Primary HCC; other cancer types, such as cholangiocellular carcinoma or mixed liver cancer, were excluded. (b) Primary HCC without other primary cancers was a requirement. (c) Primary HCC patients who died from other causes were excluded. (d) Patients with primary HCC who had undergone only surgical resection. (e) Patients with primary HCC who were unwilling to attend appointments for this research were excluded.

The original protocols were reviewed and approved by the ethical review boards of the Southwest Hospital and included consent for tissue acquisition and biomarker investigation, and all participants provided written consent.

### 2.2 Follow‐up

All patient follow‐ups were performed at the Clinical Research Center of the Institution of Hepatobiliary Surgery, Southwest Hospital. Patients were observed once every 2 months in the first 2 years after surgery and then every 3‐6 months thereafter. OS was defined as the interval between the dates of surgery and death.

### 2.3 Quantitative real‐time PCR

Fresh HCC tissues were obtained from patients who undergone surgical resection at our hospital. Total RNA was isolated using RNAiso plus (Takara 9109). Reverse transcription was performed by the PrimeScriptTM RT reagent kit with gDNA Eraser (perfect real time) (Takara, RR047A). Quantitative real‐time PCR (qPCR) was performed using TB GreenTM Premix Ex TapTM II (TliRNaseH Plus) (Takara, RR820A). The qPCR cycle parameters were as followed steps: 95°C for 30 seconds, 40 cycles at 95°C for 5 seconds and 60°C for 1 minute. The results were obtained with the CFX96TM Real‐time System 3.0 software (Applied Bio‐Rad) and further analyzed by Log2 of the qRT‐PCR value of tumors relative to the average mRNA of the tumor free tissues. GAPDH was used as a loading control. The results are shown as the fold‐change relative to the control group. The primer‐specific sequences were as follows: Ubqln2 Forward: 5′‐ AGAAAGAGGAGTTCGCGGTG‐3′, Ubqln2 Reverse: 5′‐CTGAGGTCGGTTCTGGCTTT‐3′. GAPDH Forward: 5′‐TGGCACCGTCAAGGCTGAGAA‐3′, GAPDH Reverse: 5′‐TGGTGAAGACGCCAGTGGACTC‐3′.

### 2.4 Immunohistochemistry

Pathologists were asked to confirm the tumor and nontumor tissue samples with hematoxylin‐ and eosin‐stained sections. Paraffin‐embedded tumor and adjacent normal tissue samples were sectioned (4 μm thick). After being dewaxed and rehydrated, an immunohistochemistry kit (ZSGB‐BIO, SP‐9001, SP‐9002) was then used for immunohistochemical (IHC) staining. Following these steps, incubation with methanol containing 0.3% hydrogen peroxide was performed for 30 minutes to block endogenous peroxidase activity. The sections were heated in a pressure cooker filled with the Sodium Citrate Antigen Retrieval solution (Solarbio, C1032) and incubated for 2.5 minutes after boiling with obvious steam. Natural cooling was performed, and the sections were and incubated for 30 minutes in 1% blocking serum to prevent nonspecific binding. Primary antibodies specific for Ubqln2 (Abcam, ab190283) and Ki‐67 (BD Biosciences, 550609) were diluted 1:200 and incubated with the tissue sections at 4°C overnight. Incubation with a biotinylated secondary antibody was performed for 30 minutes. Incubation with a streptavidin‐biotin complex conjugated with horseradish peroxidase was performed for 30 minutes. Diaminobenzidine (ZSGB‐BIO, ZLI‐9018) was used to develop the visualization signal. The sections were counterstained with hematoxylin.

### 2.5 Statistical analysis

SPSS 24.0 for Windows, GraphPad Prism 8.0 and R were used. Categorical variables were classified based on clinical findings. The Pearson χ^2^ test was used to analyze the relationships between Ubqln2 expression and clinicopathological features. An unpaired Student's *t* test was performed to compare differences between two continuous variables. Linear regression was used to analyze the correlation between two continuous variables, and *R* ^2^ was evaluated. Survival curves were drawn using the Kaplan‐Meier method and compared by the log‐rank test. Cox regression analysis was used for univariate and multivariate analyses. Hazard ratios (HRs) and 95% confidence intervals (95% CIs) were evaluated.

A nomogram and its calibration curve were formulated and depicted based on the results of the multivariate analysis by using the package *rms* in R version 3.6.1 (http://www.r‐project.org/). The performance of the nomogram was examined by the concordance index (C‐index).^16^ A waterfall plot was drawn by using the package *GenVisR* in R version.^17^ The column of Ubqln2 expression represents the fragments per kilobase million (FPKM) values that were also determined with R. *P *≤ .05 were considered statistically significant. All data are represented as the mean ± SEM. *P ≤ .05; **P ≤ .01; ***P ≤ .001.

## 3 RESULT

### 3.1 High expression of Ubqln2 is associated with poor clinical characteristics in HCC patients

**Figure 1:**
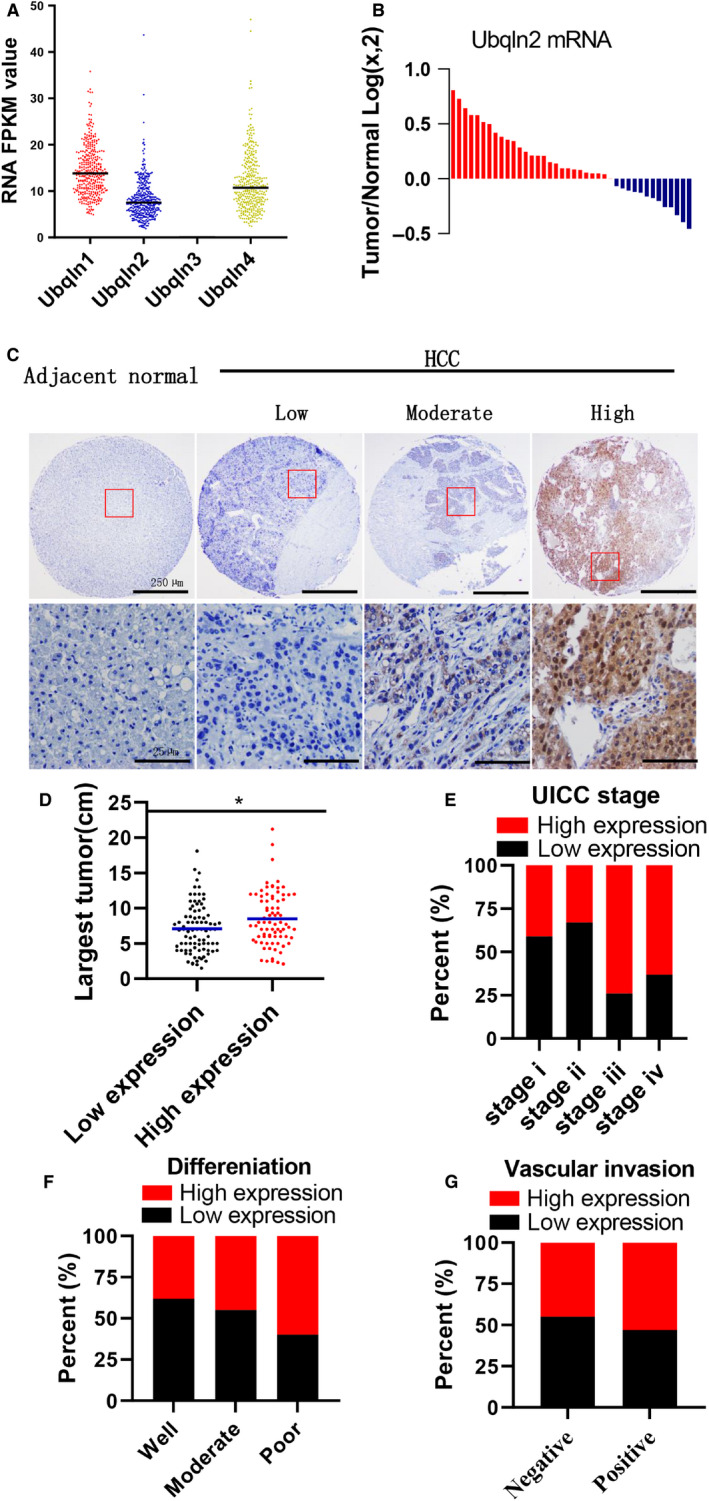
Ubqln2 labels an aggressive subset of human HCC. A, The expression of Ubqln1‐4 was analyzed in the 355 human HCC samples obtained from TCGA. B, The mRNA expression levels of Ubqln2 were evaluated in 40 HCC tissues and 3 HCC free tissues. Data represent Log2 of the qPCR value of HCC tissues relative to the 3 HCC free tissues. The red columns (sample value >0) showed that the Ubqln2 mRNA levels in the HCC tissues were (26/40) higher than the average levels in the HCC free tissues. C, Representative images shown Ubqln2 protein expression in noncancerous and primary HCC tissue samples. D, Scatter plots of the largest tumor size in patients with high or low Ubqln2 expression (unpaired *t* test, *P* = .0140). E‐G, Distributions of Ubqln2 according to UICC stage (E), tumor differentiation (F), and vascular invasion status (G), as analyzed by IHC. Images were acquired at 4× or 40× magnification; scale bar, 25 or 250 µm. Data are shown as the mean ± SEM. *P < .05

Ubqlns, including Ubqln1‐4, are a family of shuttle proteins that play significant roles in a variety of biological activities including tumorigenesis.^18^ First, 355 human HCC samples were obtained from the TCGA, and mRNA FPKM values were applied to determine the expression levels of Ubqlns (the cut‐off is the best expression cut‐off defined by the TCGA). The result indicated that Ubqln3 was not expressed in HCC tissues, while Ubqln1, Ubqln2, and Ubqln4 were highly expressed (Figure 1A). Analysis of the relationship between Ubqlns expression and clinicopathologic characteristics demonstrated that Ubqln4 has no relation with clinicopathologic characteristics in HCC patients (Table S2), and Ubqln1 was only correlated with distant metastasis (Table S1), while Ubqln2 was significantly associated with poor clinicopathologic characteristics including UICC tumor stage (Pearson χ^2^ test, *P* = .022), UICC stage (Pearson χ^2^ test, *P* = .034) as well as resection potential (Pearson χ^2^ test, *P* = .017) in the TCGA cohort (Table 1). These findings indicated that Ubqln2 may play an important role in HCC. To continually determine the role of Ubqln2 in HCC, the mRNA expression of Ubqln2 was evaluated in 40 HCC tissues and 3 HCC‐free tissues. Ubqln2 mRNA was significantly increased in 65% (26 of 40) tissues, indicated that Ubqln2 was more highly expression in HCC tissues than in normal tissues (Figure 1B). To further determine the role of Ubqln2 in HCC, a cohort of 166 paired human HCC and adjacent noncancerous liver tissue samples were evaluated and analyzed to assess the relationships between the Ubqln2 protein and clinical characteristics of HCC in high and low Ubqln2 expression groups based on IHC results (Figure 1C). We found that Ubqln2 had significant relations with tumor size (Pearson χ^2^ test, *P* = .005), UICC stage (Pearson χ^2^ test, *P* = .003) and recurrence (Pearson χ^2^ test, *P* = .009) in HCC (Table 2), and the HCC samples with high Ubqln2 expression had a larger tumor size than the HCC samples with low Ubqln2 expression (Figure 1D). Interestingly, the distribution of UICC stages showed that there was a significant difference in the expression of the Ubqln2 protein only between the HCC early stages (stage i and stage ii) and advanced stages (stage iii and stage iv) (Figure 1E), whereas there were no significant differences in differentiation and vascular invasion between the high and low Ubqln2 expression groups (Figure 1F,G). Taken together, our findings demonstrated that high expression of Ubqln2 tended to be associated with poor clinical features.

**Table 1:** Relationship between Ubqln2 mRNA expression and clinicopathologic characteristics in 355 HCC patients

Characteristics	NO patients (%)	Ubqln2		*P* value
		Low	High	
Age(y)				
≤60	169 (0.48)	136	33	.837
>60	183 (0.52)	147	36	
Data unavailable	3 (0.01)	2	1	
Gender				
Male	240 (0.68)	189	51	.295
Female	115 (0.32)	96	19	
UICC tumor stage				
T1	175 (0.49)	150	25	**.022**
T2+T3+T4	177 (0.5)	132	45	
Tx	3(0.01)	3	0	
Lymph node metastasis				
Negative	241 (0.68)	193	48	.818
Positive	3 (0.01)	2	1	
Nx	111 (0.31)	90	21	
Distant metastasis				
Negative	256 (0.72)	207	49	.786
Positive	3 (0.01)	2	1	
Mx	96 (0.27)	76	20	
UICC stage				
I	166 (0.47)	143	23	**.034**
II+III+IV	165 (0.46)	124	41	
Data unavailable	24 (0.07)	18	6	
Histological grading				
G1	53 (0.15)	43	10	.993
G2	169 (0.48)	136	33	
G3	117 (0.33)	93	24	
G4	11 (0.03)	9	2	
Gx	5 (0.01)	4	1	
Vascular invasion				
Negative	199 (0.56)	164	35	.38
Positive	102 (0.29)	81	21	
Data unavailable	54 (0.15)	40	14	
Resection status				
R0	313 (0.88)	257	56	**.017**
R1	16 (0.05)	13	3	
R2	1 (0.003)	1	0	
Rx	25 (0.07)	14	11	
Tumor states				
With tumor	109 (0.31)	84	25	.468
Tumor free	229 (0.65)	186	43	
Data unavailable	17 (0.05)	15	2	

Abbreviation: UICC, Union for International Cancer Control.

*P* < .05 was considered for statistic significance, and was marked in bold.

**Table 2:** Relationship between Ubqln2 protein expression and clinicopathologic characteristics in 166 HCC patients

Characteristics	NO patients (%)	Ubqln2		*P* value
		Low	High	
Age				
≤60	145 (0.87)	73	72	.162
>60	21 (0.13)	14	7	
Gender				
Male	147 (0.89)	79	68	.339
Female	19 (0.11)	8	11	
Tumor size				
<5 cm	49 (0.3)	34	15	**.005**
≥5 cm	117 (0.7)	53	64	
Lymph node metastasis				
Negative	158 (0.95)	85	73	.112
Positive	8 (0.05)	2	6	
Intrahepatic metastasis				
Negative	112 (0.67)	60	52	.666
Positive	54 (0.33)	27	27	
UICC stage				
I	43 (0.26)	31	12	**.003**
II	21 (0.13)	14	7	
III	75 (0.45)	32	43	
IV	27 (0.16)	10	17	
Histological grading				
G1	13 (0.08)	8	5	.231
G2	118 (0.71)	65	53	
G3	35 (0.21)	14	21	
Vascular invasion				
Negative	107 (0.64)	59	48	.343
Positive	59 (0.36)	28	31	
Recurrence				
Recurrence	107 (0.64)	48	59	**.009**
Not recurrence	59 (0.36)	39	20	

*P* < .05 was considered for statistic significance, and was marked in bold.

### 3.2 HCC patients with high expression of Ubqln2 have poor OS

To investigate the association between Ubqln2 expression and clinical prognosis, 166 HCC patients were followed. The 1‐year, 3‐year, and 5‐year OS rates were 69.88%, 42.77%, and 39.16%, respectively, for all of the patients in this study.

**Figure 2:**
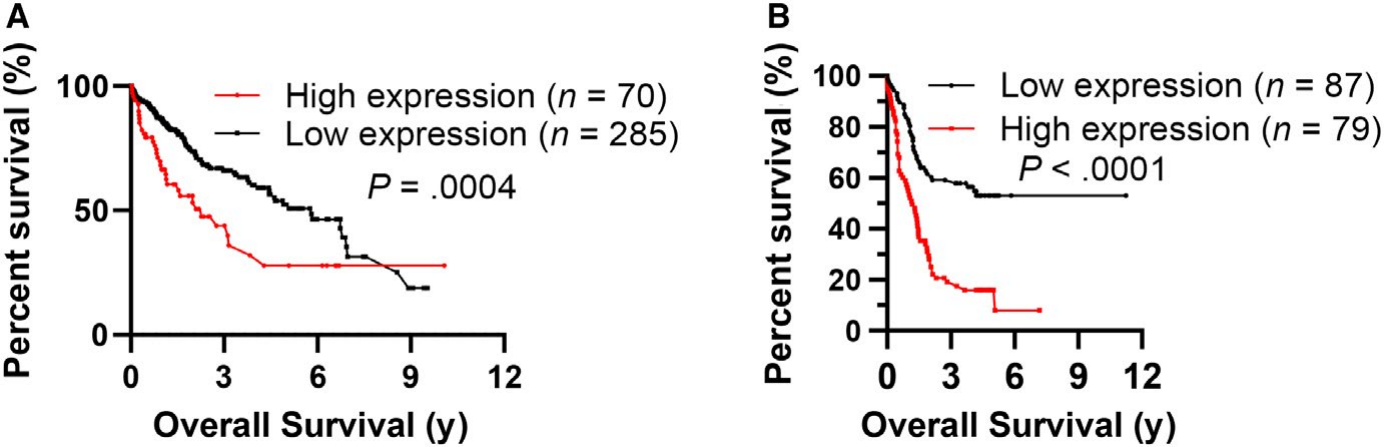
HCC patients with high expression of Ubqln2 have poor overall survival. A, Kaplan‐Meier curves for the overall survival of HCC patients from the TCGA database with high (n = 90) or low (n = 265) expression of Ubqln2. B, Kaplan‐Meier curves for the overall survival of HCC patients from Southwest Hospital with high (n = 79) or low (n = 87) intratumoral Ubqln2 staining

In the TCGA cohort, the HCC samples with high Ubqln2 mRNA expression correlated with poor OS (Figure 2A, log‐rank test, *P* = .0004). In line with this finding, Kaplan‐Meier analysis demonstrated that strong Ubqln2 staining was also significantly correlated with unfavorable OS in HCC patients (Figure 2B, log‐rank test, *P* < .0001), indicating that Ubqln2 is a key factor in the prognosis of HCC. In addition, we found that Ubqln1 did not have significant implication on HCC prognosis (Figure S1A), which further supported that Ubqln2 was a key factor in HCC prognosis.

### 3.3 Ubqln2 is an independent risk factor for prognosis in HCC

After univariate analysis of clinical variables with a Cox regression model, Ubqln2 mRNA expression level, UICC stage, UICC tumor stage, lymph node metastasis status, distant metastasis status, vascular invasion status, resection potential, and tumor status were identified as risk factors contributing to OS in the TCGA cohort (Table 3). Consistently, Ubqln2 protein expression level with other the clinical features (eg UICC stage, tumor size, intrahepatic metastasis status, vascular invasion status, and recurrence status) were also identified as risk factors contributing to OS in the IHC cohort (Table 4). Multivariate analyses demonstrated that Ubqln2 expression level, UICC stage, vascular invasion status, resection potential, and tumor status were independent risk factors for OS in the TCGA cohort (Table 3). Ubqln2 expression, histological grade, tumor size, intrahepatic metastasis status, and vascular invasion status were independent risk factors for OS in the IHC cohort (Table 4). These results indicate that high expression of Ubqln2 is an independent risk associated with poor clinical characteristics in HCC patients.

**Table 3:** Univariate and multivariate analyses indicating associations between overall survival and various risk factors in the 355 HCC patients of TCGA cohort

Univariables	n	Hazard ratio (HR)	95.0% CI	*P* value
Ubqln2 expression	285 vs 70	2.002	1.355‐2.956	**.000**
Gender	240 vs 115	0.823	0.576‐1.175	.283
Age	169 vs 183 vs 3	1.304	0.924‐1.842	.131
Histological Grading	53 vs 169 vs 117 vs 11 vs 5	1.089	0.86‐1.378	.479
UICC stage	166 vs 80 vs 82 vs 3 vs 24	1.804	1.385‐2.349	**.000**
UICC tumor stage	175 vs 177 vs 3	2.018	1.438‐2.831	**.000**
Lymph node metastasis	241 vs 3 vs 111	1.238	1.03‐1.488	**.023**
Distant metastasis	256 vs 3 vs 96	1.257	1.044‐1.514	**.016**
Vascular invasion	199 vs 102 vs 54	1.625	1.31‐2.016	**.000**
Resection status	313 vs 16 vs 1 vs 25	1.508	1.231‐1.848	**.000**
Tumor states	109 vs 229 vs 17	1.509	1.138‐2	**.004**
Multivariables				
Ubqln2 expression	285 vs 70	1.619	1.09‐2.405	**.017**
UICC stage	166 vs 80 vs 82 vs 3 vs 24	1.519	1.146‐2.014	**.004**
Vascular invasion	199 vs 102 vs 54	1.369	1.089‐1.721	**.007**
Resection status	313 vs 16 vs 1 vs 25	1.451	1.169‐1.801	**.001**
Tumor states	109 vs 229 vs 17	1.519	1.146‐2.014	**.019**

*P* < .05 was considered for statistic significance, and was marked in bold.

**Table 4:** Univariate and multivariate analyses indicating associations between overall survival and various risk factors in the 166 HCC patients of IHC cohort

Univariables	N	Hazard ratio (HR)	95.0% CI	*P* value
Ubqln2 expression	87 vs 79	2.868	1.914‐4.299	**.000**
Gender	147 vs 19	1.102	0.616‐1.973	.743
Age	166	0.978	0.959‐0.997	**.023**
Histological grading	13 vs 118 vs 35	2.353	1.609‐3.439	**.000**
UICC stage	43 vs 21 vs 75 vs 27	1.705	1.389‐2.094	**.000**
Tumor size	166	1.129	1.078‐1.181	**.000**
Lymph node metastasis	158 vs 8	1.675	0.732‐3.83	.222
Intrahepatic metastasis	112 vs 54	2.005	1.347‐2.984	**.001**
Vascular invasion	107 vs 59	2.654	1.786‐3.944	**.000**
Recurrence	107 vs 59	2.997	1.81‐4.964	**.000**
Multivariables				
Ubqln2 expression	87 vs 79	2.692	1.785‐4.058	**.000**
Histological grading	13 vs 118 vs 35	1.858	1.257‐2.748	**.002**
Tumor size	166	1.06	1.009‐1.114	**.022**
Intrahepatic metastasis	112 vs 54	1.582	1.05‐2.383	**.028**
Vascular invasion	107 vs 59	1.978	1.283‐3.049	**.002**

*P* < .05 was considered for statistic significance, and was marked in bold.

### 3.4 The expression of Ubqln2 has positive associations with proliferation markers

**Figure 3:**
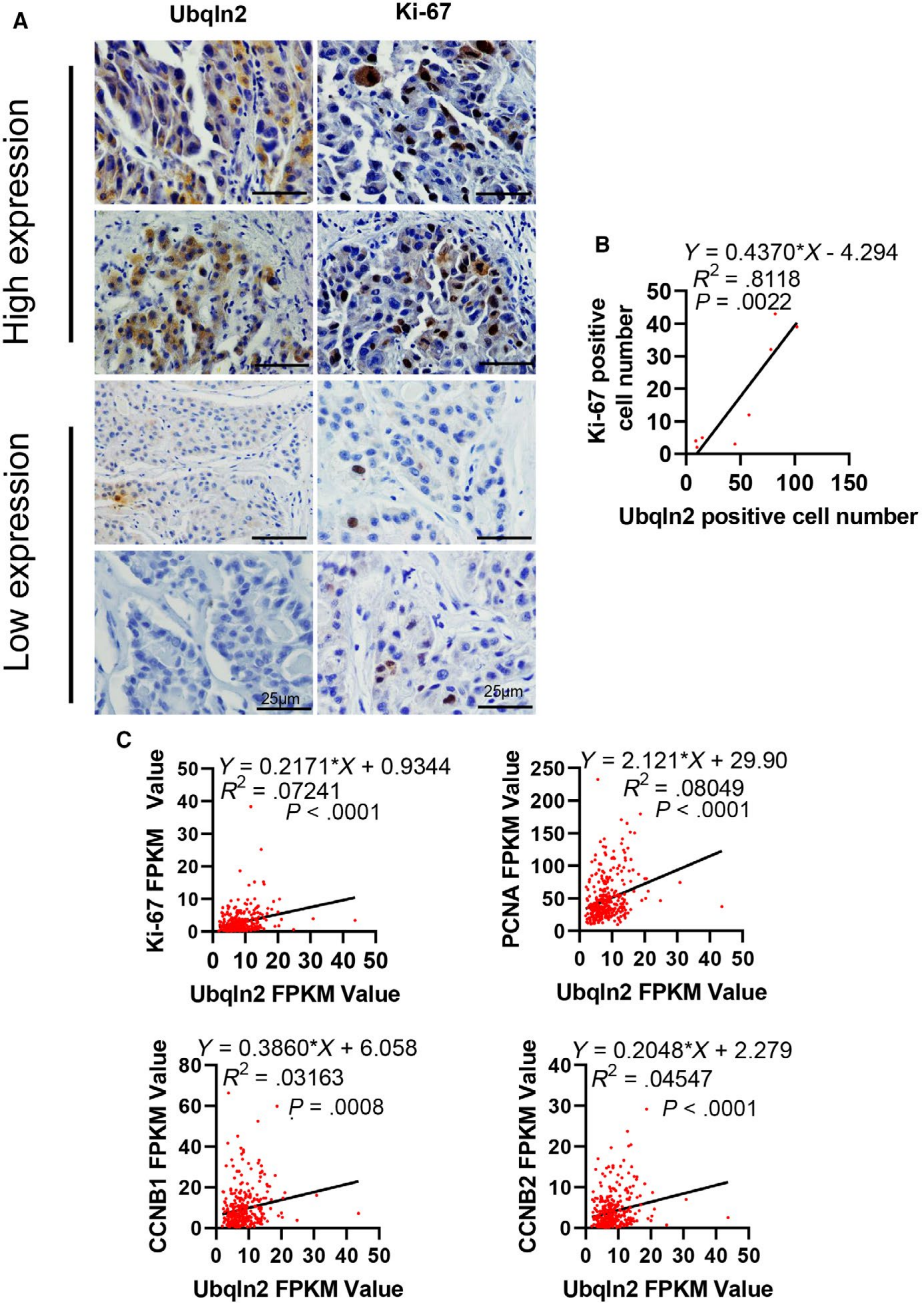
The expression of Ubqln2 has positive associations with proliferation markers. A, Representative images show that HCC samples with high expression of Ubqln2 highly expressed Ki67. B, Ubqln2 and Ki‐67 positive cells were counted in 500 cells. Lineal regression shows the positive relation between Ubqln2 and Ki‐67 (*P* = .0022). C, Linear regressions of FPKM values show the positive relations between Ubqln2 and the general proliferation markers Ki‐67 (*P* < .0001), PCNA (*P* < .0001), CCNB1 (*P* = .0008), and CCNB2 (*P* < .0001). Images were acquired at 40× magnification; scale bar, 25 µm. Data are shown as the mean ± SEM. ***P < .001

The expression of Ubqln2 was closely correlated with tumor size and UICC stage; thus, we examined the protein expression of Ubqln2 and Ki‐67 in eight samples of human HCC by IHC staining. Representative high and low expression images are shown in Figure 3A. Positive cell numbers for high and low Ubqln2 staining were calculated by counting 500 cells. Obviously, linear regression analysis shown HCC with high Ubqln2 expression tended to highly express ki‐67 (Figure 3B). The FPKM values of several general proliferation markers including Ki‐67, PCNA, CCNB1, and CCNB2 in HCC patients were downloaded from the TCGA database.^19^ Linear regression was used, and we observed that the correlations between Ubqln2 and these markers were positive (Figure 3C), indicating that the function of Ubqln2 is promoting proliferation.

### 3.5 Ubqln2 plays an important role in evaluating the prognosis of patients with HCC

**Figure 4:**
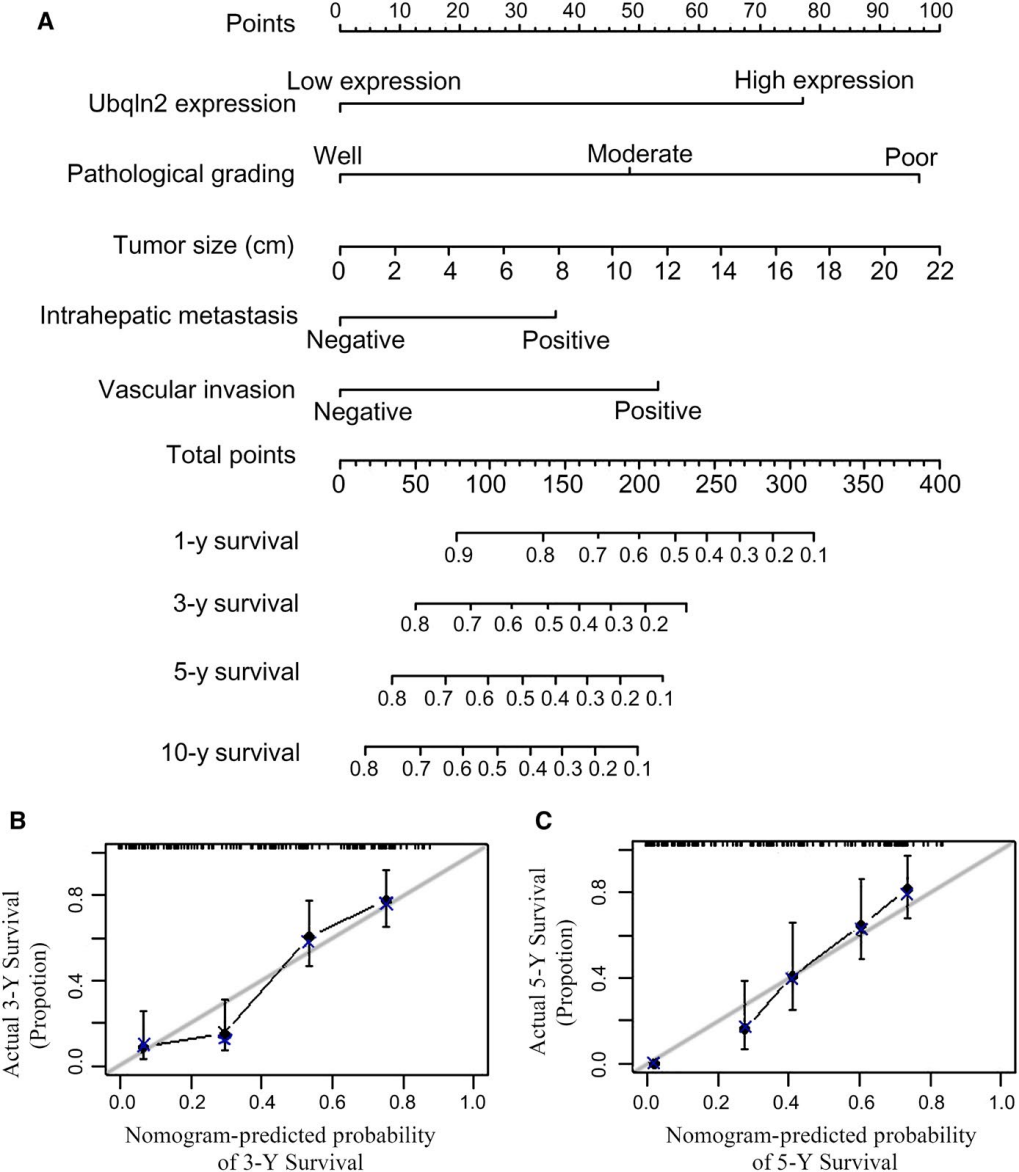
Hepatocellular carcinoma survival nomogram. A, In the nomogram, all relevant patient values are located along each variable axis, and a Points axis is drawn upward to determine the scores calculated for each variable value. The sum of the scores is located on a line called the Total Points axis, and four lines are drawn downward to the survival axes to determine the likelihood of 1‐, 3‐, 5‐, or 10‐y survival. B,C, The calibration curve for predicting patient survival at 3 y (B) and 5 y (C) in the IHC cohort is shown. The nomogram‐predicted probability of OS is plotted on the *x* axis, and actual OS is plotted on the y axis. The blue line represents the prediction, and the gray line represents the ideal

To investigate the role of Ubqln2 in evaluating the prognosis of patients with HCC, 166 samples from patients with HCC were further analyzed. According to the results of multivariate analyses with the Cox regression model, a prognostic nomogram that integrated all significant independent factors for OS was created and is shown in Figure 4A. The C‐index for OS prediction was 0.745. High expression of Ubqln2 contributed approximately 78 points to the total points, which was more points than those contributed by moderate pathological stage, tumor size less than 10 cm, intrahepatic metastasis and vascular invasion, indicating that Ubqln2 plays a more important role than the other risk factors in the prognosis of patients with HCC. The calibration plot for the probabilities of survival at 3 and 5 years after surgery showed optimal agreement between the prediction by the nomogram and actual observations (Figure 4B,C).

### 3.6 The expression of Ubqln2 is associated with mutated CTNNB1

**Figure 5:**
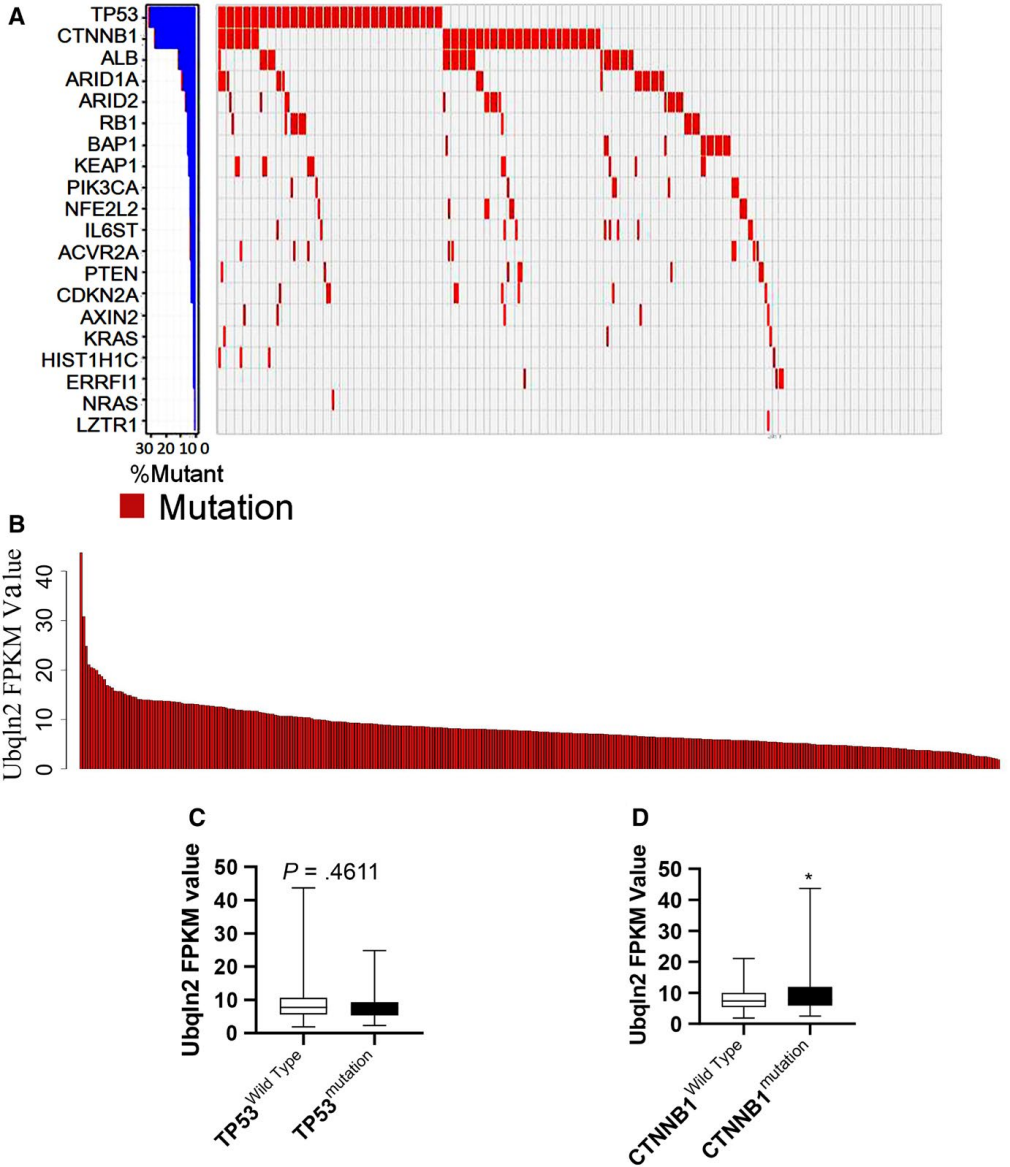
The genomic landscape of HCC and mutational signatures. A, The waterfall plot shows the top 20 most commonly mutated genes in patients with HCC. The mutation frequency is listed in the bar chart on the left, and mutation types are indicated in the legend on the right. B, The bar chart shows the expression of Ubqln2 in 350 HCC patients. C,D, Differential expression of Ubqln2 existed only for CTNNB1, not for TP53

TP53, CTNNB1, ALB, AXIN2, KEAP1, BAP1, NFE2L2, LZTR1, RB1, PIK3CA, KRAS, IL6ST, CDKN2A, ARID2, ARID1A, ACVR2A, NRAS, HISR1H1C, PTEN, and ERRFI1 are the main genes that are mutated during hepatocarcinogenesis.^17^ To explore the relationships between Ubqln2 expression and genetic alterations, the mutated genes in 350 cases of HCCs obtained from the TCGA database were further analyzed (Figure 5A). Figure 5B showed the FPKM values for Ubqln2 in these cases. After comparing the wild‐type group and the mutated group for these cases, there was different expression for only the CTNNB1 gene (mutation ratio: 27.14%, Figure 5D), not the TP53 gene (mutation ratio: 30.57%, Figure 5C), indicating that the function of Ubqln2 is dependent on CTNNB1.

## 4 DISCUSSION

HCC, which constitutes the majority of liver cancer cases, is commonly considered the worst result of chronic infection with HBV or HCV, aflatoxin‐contaminated foodstuff consumption, heavy alcohol intake, obesity, smoking, and type 2 diabetes.^2^ As effective diagnostic markers are limited, the detection of HCC in the early stage is poor.^10^ The multitarget inhibitor sorafenib can prolong the survival of advanced‐stage HCC patients by approximately 3 months.^20^ However, it has not yet met expectations. Thus, other analogs were developed in succession.^3^, ^21^ Due to drug resistance and adverse effects, curative efficacy is still barely satisfactory.^7^, ^8^

The UPS plays pivotal roles in essential cellular functions in eukaryotes through timely degradation of various regulatory proteins.^22^ In this study, we demonstrated increased clinical risk in the HCC group with high Ubqln2 expression, and we developed a model to evaluate prognostic survival via assessment of the Ubqln2 expression level. Ubqln2, a member of the Ubqlns, is a proteasomal shuttle protein and, with the addition of E1, E2, E3, and DUBs, constitutes the UPS. E1 activates ATP‐dependent ubiquitin and then transfers it to E2. E2 ultimately combines with E3 to catalyze the combination of ubiquitin and substrates.^10^, ^23^ The shuttle proteins have two main domains: an N‐terminal ubiquitin‐like domain (UBL), which interacts with the proteasome, and a C‐terminal ubiquitin‐associating domain (UBA), which interacts with ubiquitin and polyubiquitin.^12^ The entire process can be reversed by DUBs.^24^

In previous studies, Ubqln2 appeared to be a risk factor for osteosarcoma and a novel marker for detecting urothelial carcinoma cells in urine.^14^, ^25^ In our study, HCC patients with high expression of Ubqln2 had reduced OS, indicating that Ubqln2 can lead to a poor prognosis. Moreover, the expression level of Ubqln2 was intimately associated with tumor size and UICC stage in HCC patients at both the protein and mRNA levels, suggesting that Ubqln2 is a potential activator that promotes tumor growth. Consistent with our finding, high expression of Ubqln2 was closely correlated with the expression of some proliferation markers, such as Ki‐67, PCNA, CCNB1, and CCNB2.

Nomograms have been shown to be relatively accurate in predicting prognosis in some cancers.^16^ Thus, a prognostic nomogram for HCC patients was constructed. Surprisingly, high Ubqln2 expression contributed approximately 78 points to survival prediction, which was much higher than the points contributed by many other features, indicating that HCC patients with high expression of Ubqln2 are apt to have a poor prognosis and short survival and hinting that inhibition of Ubqln2 may greatly reduce risks. These results demonstrated that blockade of Ubqln2 is a strong candidate to improve survival in this group.

Moreover, due to high therapeutic resistance and the difficulty in treating HCC, novel therapies for HCC still remain an unmet medical need. Thus, whole‐exome sequencing has been performed, and frequently mutated genes in HCC have been identified.^4^, ^17^ We examined the relationships between the expression of Ubqln2 and frequently mutated genes. Both TP53 and CTNNB1 are TERT promoters and the most frequently mutated genes^26^; however, Ubqln2 expression seemed to differ only between mutated and wild‐type CTNNB1 cases. CTNNB1 encodes β‐catenin.^27^ β‐Catenin is a key coactivator downstream of oncogenic Wnt signaling, so mutated CTNNB1 is considered oncogenic.^28^ Aberrantly activated Wnt/β‐catenin signaling plays a pivotal role in the malignant transformation of liver cells and malignant expansion of cancer cells.^29^ CTNNB1 and TP53 mutations represent two different tumor phenotypes,^30^ and mutated CTNNB1 has a significant statistical association with high expression of Ubqln2, suggesting that Ubqln2 may play an important role in mutated CTNNB1‐related signaling.

In summary, high Ubqln2 expression labels a group of HCC patients with aggressive disease, and Ubqln2 has close correlations with tumor size and UICC stage. HCC patients with high expression of Ubqln2 are predicted to have poor survival. Ubqln2 seems to have a close relationship with mutated CTNNB1. Based on clinical research, our second aim is to clarify the mechanism by which Ubqln2 functions in HCC and determine the role of Ubqln2 in HCC with mutated CTNNB1.

## CONFLICT OF INTEREST

The authors declare no potential conflict of interest.

## AUTHOR CONTRIBUTIONS

Chuan‐Ming Xie, Ping Bie, and Yuan‐Deng Luo were involved in conception and design. Chuan‐Ming Xie was involved in administrative support and writing—review and editing. Chuan‐Ming Xie and Geng Chen were involved in financial support. Yuan‐Deng Luo was involved in data analysis and interpretation, and writing—initial draft. Yuan‐Deng Luo, Hong‐Qiang Yu, Xiao‐Yu Liu, Deng Huang, Hai‐Su Dai, Lei Fang, Yu‐Jun Zhang, Jie‐Juan Lai, Yan Jiang, Ling Shuai, and Lei‐Da Zhang were involved in collection and assembly of data. All authors gave final approval of manuscript and accountable for all aspects of the work.

## Supporting information

Supplementary Material

## Data Availability

All data, models, and code generated or used during the study appear in the submitted article.
