# Supplementary material for: Prognostic and predicted significance of Ubqln2 in patients with hepatocellular carcinoma
Source: Cancer Med. 2020 Apr 15;9(12):4083–94. doi: 10.1002/cam4.3040 (PMC7300399; doi:10.1002/cam4.3040)
Supplement: Supplementary file 1 — Supplementary Material [file CAM4-9-4083-s001.pdf]

Figure S1

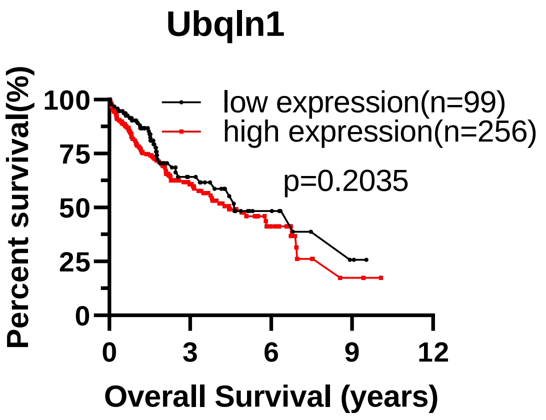
Ubqln1
Percent survival(%)
100
75
50
25
0
low expression(n=99)
high expression(n=256)
p=0.2035
0
3
6
9
12
Overall Survival (years)

Table S1. Relationship between Ubqln1 protein expression and clinicopathologic characteristics in 355 HCC patients

Characteristics	N.O. patients (%)	Ubqln1 Low	Ubqln1 High	P value
Age(yrs)				
≤60	169(0.48)	48	121	0.554
>60	183(0.52)	51	132	
Data unavailable	3(0.01)	0	3	
Gender				
Male	240(0.68)	70	170	0.437
Female	115(0.32)	29	86	
UICC tumor stage				
T1	175(0.49)	56	119	0.219
T2+T3+T4	177(0.5)	42	135	
Tx	3(0.01)	1	2	
Lymph node metastasis				
Negative	241(0.68)	65	176	0.433
Positive	3(0.01)	0	3	
Nx	111(0.31)	34	77	
Distant metastasis				
Negative	256(0.72)	70	186	**0.02**
Positive	3(0.01)	3	0	
Mx	96(0.27)	26	70	
UICC stage				
I	166(0.47)	54	112	0.155
II+III+IV	165(0.46)	38	127	
Data unavailable	24(0.07)	7	17	
Histological grading				
G1	53(0.15)	13	40	0.616
G2	169(0.48)	52	117	
G3	117(0.33)	28	89	
G4	11(0.03)	4	7	
Gx	5(0.01)	2	3	
Vascular invasion				
Negative	199(0.56)	56	143	0.91
Positive	102(0.29)	30	72	
Data unavailable	54(0.15)	13	41	
Resection status				
R0	313(0.88)	87	226	0.448
R1	16(0.05)	4	12	
R2	1(0.003)	1	0	
Rx	25(0.07)	7	18	
Tumor states				
With tumor	109(0.31)	22	87	0.773
Tumor free	229(0.65)	71	158	
Data unavailable	17(0.05)	6	11	

Table S2. Relationship between Ubqln4 protein expression and clinicopathologic characteristics in 355 HCC patients

Characteristics	N.O. patients (%)	Ubqln4 Low	Ubqln4 High	P value
Age(yrs)				
≤60	169(0.48)	75	94	0.235
>60	183(0.52)	87	96	
Data unavailable	3(0.01)	0	3	
Gender				
Male	240(0.68)	106	134	0.423
Female	115(0.32)	56	59	
UICC tumor stage				
T1	175(0.49)	84	91	0.485
T2+T3+T4	177(0.5)	76	101	
Tx	3(0.01)	2	1	
Lymph node metastasis				
Negative	241(0.68)	104	137	0.326
Positive	3(0.01)	1	2	
Nx	111(0.31)	57	54	
Distant metastasis				
Negative	256(0.72)	115	141	0.804
Positive	3(0.01)	1	2	
Mx	96(0.27)	46	50	
UICC stage				
I	166(0.47)	82	84	0.361
II+III+IV	165(0.46)	71	94	
Data unavailable	24(0.07)	9	15	
Histological grading				
G1	53(0.15)	27	26	0.087
G2	169(0.48)	86	83	
G3	117(0.33)	43	74	
G4	11(0.03)	4	7	
Gx	5(0.01)	2	3	
Vascular invasion				
Negative	199(0.56)	90	109	0.409
Positive	102(0.29)	51	51	
Data unavailable	54(0.15)	21	33	
Resection status				
R0	313(0.88)	145	168	0.355
R1	16(0.05)	8	8	
R2	1(0.003)	1	0	
Rx	25(0.07)	8	17	
Tumor states				
With tumor	109(0.31)	45	64	0.344
Tumor free	229(0.65)	107	122	
Data unavailable	17(0.05)	10	7	
